# Retraction: Hepatitis C Virus Core Protein Down-Regulates p21^Waf1/Cip1^ and Inhibits Curcumin-Induced Apoptosis through MicroRNA-345 Targeting in Human Hepatoma Cells

**DOI:** 10.1371/journal.pone.0235375

**Published:** 2020-06-24

**Authors:** The PLOS ONE Editors

After this article [1] and its subsequent correction [2] were published, additional concerns were raised:

- The *PLOS ONE* Editors re-reviewed the concerns about the 4th and 5th FACS panels in Fig 3C, which were discussed previously in [2]. Based on our reassessment, we do not consider that the prior correction [2] resolves the concerns about similarities between the lower quadrants of these panels. We regret that this was not fully addressed before the correction was published. The authors provided additional dot plots for four of the five experimental conditions but noted that the raw data (.fcs files) are no longer available. As such, the concerns about this figure remain unresolved and call into question the validity of the results reported for the 25 and 50 nM miR-345 mimic conditions.
- The β-actin panels in Fig 2C and S4 Fig appear similar, although they represent different experiments. This image issue has implications for the quantitative data reported for the corresponding western blot panels. The authors provided results from an independent replicate for each experiment and noted that an error may have been made in preparing S4 Fig, but they did not provide uncropped images of the original raw blots to support the published results.
- In the original version of Fig 4B [1], the lower edge of the HA-Core173 panel and the upper edge of the HA-Core173 + miR-345 inhibitor (5 nM) panels align in a manner suggesting that the two panels may show neighboring fields of the same data. In addition, as noted in [2], duplicate images were shown in Fig 4B as representing HA-Core191 + miR-345 inhibitor (10nM) and HA-Core173 + miR-345 inhibitor (10nM). The authors confirmed these issues and noted they may have resulted from mislabeling of images and/or errors in figure preparation. In the previous correction [2], Fig 4B images were replaced with replicate data. The authors provided image data in support of the original published results and replication results but these did not resolve the concerns about the original figure.
- The β-actin blots are overexposed in Figs 3A, 4A and 4B, such that they do not provide a clear representation of the relative protein abundance across lanes. This calls into question the reliability of quantitative results reported for the corresponding western blot experiments. The authors provided replication results for each of these experiments along with associated quantitative data, but did not provide uncropped images of the original raw blots reported in the the published figures. The quantitative data provided relied on the saturated β-actin blots, did not resolve the concern about the reliability of the densitometric analyses, and indicated that the published densitometry results represent results from a single sample per condition rather than summarizing data across multiple experimental replicates.
- The 6.25 and 12.5 μM Curcumin panels shown for the Annexin V-FITC experiment in S4 Fig appear similar when brightness is adjusted. The authors commented that this was due to an error in figure preparation and they provided results from replicate experiments to support this figure.

The above concerns call into question the reliability of the reported results and conclusions, and so the *PLOS ONE* Editors retract this article.

The corresponding author (TYH) notified the journal that TYS and TYH agreed with retraction and apologize for the issues with the published article, but stand by the article’s findings.

The other authors either could not be reached or did not respond directly.
